# Comparison of fully-automated radiosyntheses of [^11^C]erlotinib for preclinical and clinical use starting from in target produced [^11^C]CO_2_ or [^11^C]CH_4_

**DOI:** 10.1186/s41181-018-0044-1

**Published:** 2018-05-30

**Authors:** Cécile Philippe, Severin Mairinger, Verena Pichler, Johann Stanek, Lukas Nics, Markus Mitterhauser, Marcus Hacker, Thomas Wanek, Oliver Langer, Wolfgang Wadsak

**Affiliations:** 1 Department of Biomedical Imaging and Image-guided Therapy, Division of Nuclear Medicine, Medical University of Vienna, Vienna, Austria; 2 Department of Pharmaceutical Technology and Biopharmaceutics, University of Vienna, Vienna, Austria; 3 Biomedical Systems, Center for Health & Bioresources, AIT Austrian Institute of Technology GmbH, Seibersdorf, Austria; 4 Department of Nutritional Sciences, University of Vienna, Vienna, Austria; 5 Ludwig Boltzmann Institute Applied Diagnostics, Vienna, Austria; 6 Department of Clinical Pharmacology, Medical University of Vienna, Vienna, Austria; 7 CBmed, Graz, Austria; 8 Department of Inorganic Chemistry, University of Vienna, Vienna, Austria

**Keywords:** [^11^C]erlotinib, Radiosynthesis, PET, EGFR, Tyrosine kinase inhibitor, HPLC, Quality control

## Abstract

**Background:**

[^11^C]erlotinib has been proposed as a PET tracer to visualize the mutational status of the epidermal growth factor receptor (EGFR) in cancer patients. For clinical use, a stable, reproducible and high-yielding radiosynthesis method is a prerequisite. In this work, two production schemes for [^11^C]erlotinib applied in a set of preclinical and clinical studies, starting from either [^11^C]CH_4_ or [^11^C]CO_2_, are presented and compared in terms of radiochemical yields, molar activities and overall synthesis time. In addition, a time-efficient RP-HPLC method for quality control is presented, which requires not more than 1 min.

**Results:**

[^11^C]erlotinib was reliably produced applying both methods with decay-corrected radiochemical yields of 13.4 ± 6.2% and 16.1 ± 4.9% starting from in-target produced [^11^C]CO_2_ and [^11^C]CH_4_, respectively. Irradiation time for the production of [^11^C]CO_2_ was higher in order to afford final product amounts sufficient for patient application. Overall synthesis time was comparable, mostly attributable to adaptions in the semi-preparative HPLC protocol. Molar activities were 1.8-fold higher for the method starting from [^11^C]CH_4_ (157 ± 68 versus 88 ± 57 GBq/μmol at the end of synthesis).

**Conclusions:**

This study compared two synthetic protocols for the production of [^11^C]erlotinib with in-target produced [^11^C]CO_2_ or [^11^C]CH_4_. Both methods reliably yielded sufficiently high product amounts for preclinical and clinical use.

## Background

The epidermal growth factor receptor (EGFR) belongs to the family of receptor tyrosine kinases and is one of the most frequently overexpressed proteins in malignant tumors (Ciardiello and Tortora 2008). Therefore, it has become an attractive target for cancer treatment. In this regard, EGFR specific antibodies such as cetuximab (Erbitux®) as well as small molecule tyrosine kinase inhibitors (TKIs) such as gefitinib (Iressa®) and erlotinib (Tarceva®, OSI-774) have been developed. Erlotinib reached marketing authorization in 2004 and belongs to the first-generation reversible TKIs (Cohen et al. 2005). It is used to treat non-small cell lung cancer (NSCLC) and pancreatic cancer (Singh and Jadhav 2017). One crucial parameter for prediction of treatment response is the mutational status of the EGFR. In order to identify the subset of patients who will benefit from therapy with erlotinib, positron emission tomography (PET) with [^11^C]erlotinib has been proposed (Memon et al. 2009; Bahce et al. 2013; Petrulli et al. 2013; Slobbe et al. 2015). Owing to the short radioactive half-life of carbon-11 (t_1/2_ = 20.4 min), a short overall synthesis time is desirable as it determines absolute radiochemical yields and molar activities. Hence, every adaption in the radiosynthesis protocol reducing the synthesis time significantly improves the outcome. Additionally, depending on the intended use of the radiotracer (i.e. preclinical or clinical), different approaches for formulation of the final product may be required. For example, higher doses in an overall larger volume are applied in patients, whereas in small animal studies the volume of injection is very limited and therefore, a higher concentration of the PET tracer is required.

The present study focused on comparing the radiosynthesis of [^11^C]erlotinib starting from either [^11^C]CO_2_ or [^11^C]CH_4_ in terms of time efficiency, overall yields and molar activity. The synthesis was conducted according to Bahce et al. (2013) and was effectively implemented for preclinical and clinical studies (Traxl et al. 2017; Bauer et al. 2017). Additionally, a time-efficient HPLC method for quality control (Nics et al. 2018) was applied to minimize post-production loss of [^11^C]erlotinib caused by a time intensive quality control and subsequent decline of molar activity.

## Methods

### Materials

Unless otherwise stated all chemicals were purchased from Sigma-Aldrich Chemie (Schnelldorf, Germany) or Merck (Darmstadt, Germany) at analytical grade and were used without further purification. The Ni catalyst (Shimalilte Ni reduced, 80/100 mesh) was purchased from Shimadzu (Kyoto, Japan). The precursor 6-*O*-desmethyl-elotinib (OSI-420; GMP grade) was purchased from Syncom B.V. (Groningen, Netherlands) and the reference compound erlotinib (*N*-(3-ethinylphenyl)-6,7-bis(2-methoxyethoxy)-quinazolin-4-amine) was obtained from Apollo Scientific (Bredbury, UK). Semi-preparative high performance liquid chromatography (HPLC) column (Chromolith® SemiPrep RP-18e, 100–10 mm; guard column: 10–10 mm) and analytical HPLC column (Chromolith Performance RP-18e, 100–4.6 mm; guard column: 10–4.6 mm) were purchased from Merck (Darmstadt, Germany). The analytical HPLC column for the optimized quality control (XBridge® Shield RP-18; 2.5 μm; 3.0–50 mm) and C18plus SepPak® cartridges for solid phase extraction (SPE) were purchased from Waters (Waters® Associates Milford, MA, USA). Low-protein binding Millex GS® 0.22 μm sterile filters were purchased from Millipore® (Bedford, MA, USA). Gas chromatography (GC) capillary column (forte GC Capillary Column ID-BP20; 12 m × 0.22 mm × 0.25 μm) was obtained from SGE Analytical Sciences Pty Ltd. (Victoria, Australia).

### Instrumentation

[^11^C]CO_2_ (further referred to as method 1) and [^11^C]CH_4_ (method 2) were produced in two separate GE PET trace cyclotrons (General Electric Medical System, Uppsala, Sweden) via the ^14^N(p, α)^11^C nuclear reaction by irradiation of a gas target (Aluminium) filled with N_2_ + 1% O_2_ or N_2_ + 10% H_2_, respectively. Typical beam currents were 48–65 μA (for [^11^C]CO_2_) or 25 μA (for [^11^C]CH_4_) and the irradiation was stopped as soon as the desired activity level was reached (approx. 80–130 GBq [^11^C]CO_2_ or 27–43 GBq [^11^C]CH_4_; corresponding to 30–40 min or 12–22 min irradiation time). The syntheses of [^11^C]erlotinib starting from either [^11^C]CO_2_ or [^11^C]CH_4_] were performed in two different TRACERlab™ FX C Pro synthesis modules (GE Healthcare, Uppsala, Sweden). Conventional analytical HPLC was performed using an Agilent 1260 system (Agilent Technologies GmbH, Santa Clara, CA, USA) equipped with a UV-detector, a BGO detector (Elysia-Raytest, Straubenhardt, Germany) and controlling software Agilent Chemstation or Elysia-Raytest GINA Star. Optimized analytical HPLC for the 1 min quality control run was performed as described elsewhere (Nics et al. 2018). For the optimization, an analytical HPLC column, X-Bridge BEH Shield RP-18, 4.6 × 50 mm, 2.5 μm, 130 Å (Waters GmbH), was used. The analytical HPLC analyses was performed on a single Agilent 1260 system equipped with a quaternary pump (G1311B), a multi wavelength UV-detector (G1365D), a column oven (G1316A), a manual injector (G1328C), a NaI (Tl) detector from Berthold Technologies (Bad Wildbad, Germany) and GINA Star controlling software (Elysia-Raytest; Straubenhardt, Germany). The osmolality was measured using a Wescor osmometer Vapro® 5600 (Sanova Medical Systems, Vienna, Austria) and pH was measured using a WTW inoLab 740 pH meter (WTW, Weilheim, Germany). Gas chromatography was performed using a 430-GC system (Bruker Daltonik GmbH, Bremen, Germany).

### Preparation of the synthesis module

**Fig. 1:**
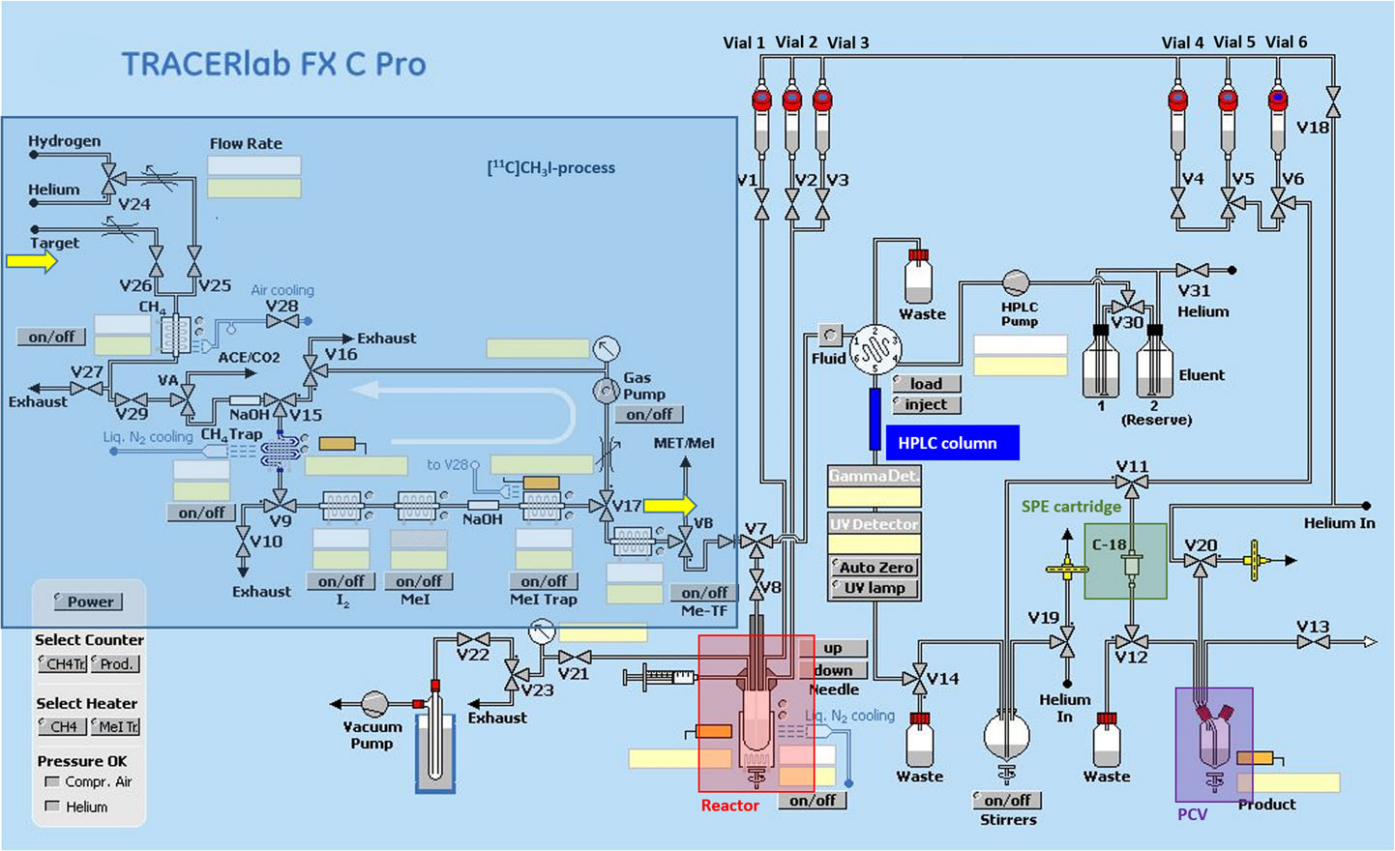
Scheme of the commercial ^11^C-synthesizer used for the radiosynthesis and purification of [^11^C]erlotinib (SPE: solid phase extraction; PCV: product collection vial)

A scheme of the synthesis module is presented in Fig. 1. All parts prior to HPLC-purification (reactor, HPLC injector, tubing) were rinsed with water and acetone and then dried with a stream of helium. Parts after semi-preparative HPLC (i.e. solid-phase extraction (SPE) tubing, dilution flask, product collection vial, product outlet tubing) were cleaned using ethanol and water.

### Fully-automated radiosynthesis of [^11^C]erlotinib

**Fig. 2:**
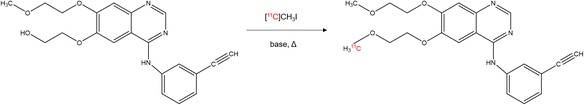
Radiosynthesis of [^11^C]erlotinib

**Fig. 3:**
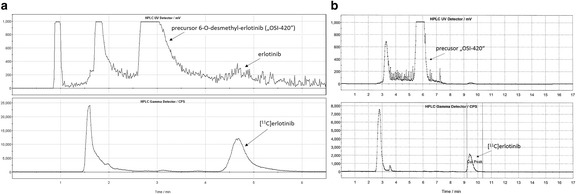
Semi-preparative chromatogram of the reaction solution of [^11^C]erlotinib (top: UV-channel; bottom: radioactivity-channel) for method 1 (**a**) and method 2 (**b**). Note that flow rate was 8 mL/min for method 1 and 2.5 mL/min for method 2

[^11^C]erlotinib was synthesized by reacting 6-*O*-desmethyl-erlotinib with [^11^C]CH_3_I (Fig. 2), which was produced either from [^11^C]CO_2_ (target filled with N_2_ + 1% O_2_) or directly from [^11^C]CH_4_ (target filled with N_2_ + 10% H_2_). In method 1, [^11^C]CO_2_ was trapped on a molecular sieve (4 Å) and consequently reduced to [^11^C]CH_4_ over a Ni catalyst with hydrogen at 400 °C, while in method 2 the Ni catalyst furnace was bypassed since [^11^C]CH_4_ was directly produced in the cyclotron. The [^11^C]CH_4_ was trapped in a cooled PorapakQ column and was further processed to [^11^C]CH_3_I by standard procedure via the gas phase method (Larsen et al. 1997). Subsequently, [^11^C]CH_3_I was released and transferred into the reactor containing the precursor solution (0.8–1.0 mg, 1.9–2.4 μmol 6-*O*-desmethyl-erlotinib) dissolved in 150 μL CH_3_CN and 3 μL TBAH solution (54.0–56.0% (*w*/*v*) in H_2_O) at 25 °C. The reactor was sealed and heated to 75 °C for 5 min. After cooling down to room temperature, the reaction mixture was quenched by addition of 0.6 mL HPLC solvent and subsequently transferred into the built-in HPLC system. Semi-preparative HPLC was performed with CH_3_CN/NH_4_OAc buffer (0.2 M, pH 5.0) (65/35, *v*/v) at flow rates of 8 mL/min (method 1) or 2.5 mL/min (method 2). The HPLC eluate was monitored in series for radioactivity and ultraviolet (UV) absorption at a wavelength of 254 nm. On this system, radiolabelling precursor 6-*O*-desmethyl-erlotinib and product [^11^C]erlotinib eluted with retention times of 2.5–3.5 min and 4.5–5.5 min (method 1, Fig. 3a) and 5.2–6.5 min and 9–10 min (method 2, Fig. 3b), respectively. The [^11^C]erlotinib product fraction was collected in a bulb and diluted with 100 mL of sterile water. The resulting solution was then pushed through a preconditioned C18 SPE cartridge (10 mL EtOH, 20 mL H_2_O, dried). The cartridge was washed with 10 mL of water and the purified product was eluted with 1.5 mL of ethanol into the product vial.

For preclinical use, the EtOH was then removed on a rotary evaporator and the product formulated in in 0.9% aq. saline/0.1 M HCl (100/0.1, *v*/v) at an approximate concentration of 370 MBq/mL for intravenous injection.

For clinical use, the SPE cartridge was washed with 5 mL 0.9% saline solution, which was collected into the product vial containing 6 mL phosphate-buffer saline (PBS) solution. The resulting solution was transferred through a 22 μm sterile filter into a sterile 25 mL vial containing further 5 mL of 0.9% saline solution. Hence, the final total volume of the product solution was 17.5 mL (containing 8.6% ethanol).

### Quality control of [^11^C]erlotinib

**Fig. 4:**
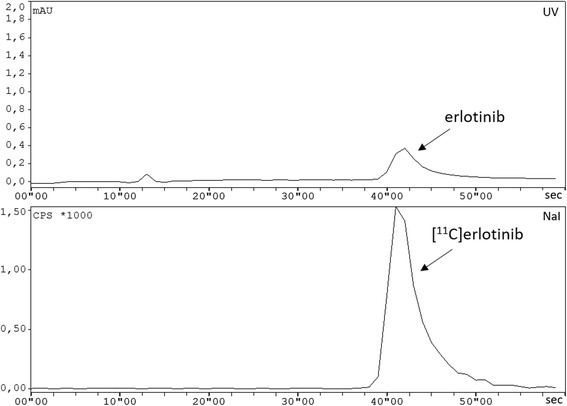
Typical chromatogram of the purified and formulated [^11^C]erlotinib using the optimized analytical HPLC (top: UV-channel; bottom: radioactivity-channel)

Chemical and radiochemical impurities were detected and quantified using analytical radio-HPLC according to the monograph in the European Pharmacopoeia (Radiopharmaceutical Preparations, 8.0/0125 2008). Radiochemical purity and molar activity of [^11^C]erlotinib were determined by analytical radio-HPLC (gradient from 0 to 15 min with 30–55% CH_3_CN in NH_4_OAc buffer (0.2 M, pH 5.0) at a flow rate of 1 mL/min. UV detection was performed at a wavelength of 334 nm. The retention time of [^11^C]erlotinib was about 8.5 min on this HPLC system. In order to accelerate the product release process, a time optimized analytical HPLC analysis was set up (mobile phase: 60% NH_4_OAc buffer (0.2 M, pH 5.0), 40% CH_3_CN, flow rate: 1 mL/min, wavelength: 334 nm). This HPLC analysis was completed within 1 min; the retention time of the precursor was 25 s (k’ = 1.08) and 46 s (k’ = 2.83) for the product [^11^C]erlotinib (Nics et al. 2018). The chemical identity of [^11^C]erlotinib was determined by co-injection of the unlabelled reference compound erlotinib. Molar activity was calculated by determining the mass of erlotinib in the final product solution (UV-channel). For calculation of radiochemical purity (radioactivity channel), the percentage of [^11^C]erlotinib relative to total radioactivity was determined (threshold ≥95%). Sample chromatograms are given in Fig. 4. Residual solvents were analysed by GC. Osmolality and pH were determined to assure safe administration using standard methods. Radionuclidic purity was assessed by recording of the corresponding gamma spectrum and additional measurement of the physical half-life. Testing of sterility and concentration of bacterial endotoxins was performed using standard protocols at the Department of Infection Diseases and Tropical Medicine (Medical University of Vienna, Austria).

## Results

The fully-automated synthesis and purification of [^11^C]erlotinib was performed successfully and with high reliability within approximately 35 min after the end of bombardment (EOB) on a GE FX C Pro module starting either from [^11^C]CO_2_ (method 1) or [^11^C]CH_4_ (method 2). All values are given by arithmetic means ± standard deviation.

Method 1: In 94 runs, 2.6 ± 1.3 GBq of [^11^C]erlotinib were obtained as a sterile solution ready for application (decay-corrected radiochemical yields are 8.5 ± 4.3% and 13.4 ± 6.2% based on [^11^C]CO_2_ and trapped [^11^C]CH_3_I, respectively). Three syntheses failed due to technical problems, especially clogging of transfer lines. Radiochemical purity exceeded 99% as determined by radio-HPLC. No precursor was found in the final product (limit of quantification 30.92 ng/mL (Nics et al. 2018)). The molar activity at the end of synthesis (EOS) was 88 ± 57 GBq/μmol (range: 12–264 GBq/μmol, erlotinib content in formulated solution: 0.11–4.04 μg/mL or 0.28–10.28 nmol/mL). A typical analytical HPLC chromatogram is shown in Fig. 4.

Method 2: In 35 runs, 0.76 ± 0.27 GBq of [^11^C]erlotinib were obtained (decay-corrected radiochemical yields are 7.3 ± 2.8% and 16.1 ± 4.9% based on [^11^C]CH_4_ and trapped [^11^C]CH_3_I, respectively). Radiochemical purity of [^11^C]erlotinib was greater than 98% and molar activity at EOS was 157 ± 68 GBq/μmol (range:: 65–396 GBq/μmol, 2.2–18.8 nmol erlotinib).

Typical loss during final sterile filtration was <10% of the product activity. Residual acetonitrile as determined by GC was found to be <20 ppm. Osmolality was 303 ± 21 mosmol/kg and pH was 7.5 ± 0.1. Concentration of endotoxins was found below 1.0 EU/mL and all samples passed the test for sterility. All quality parameters were in accordance with the standards for parenteral human application. The entire quality control process (except for tests for residual solvents, endotoxins and sterility) was completed within 3 min using the optimized analytical HPLC system.

A comparison of the productions starting from either [^11^C]CO_2_ or [^11^C]CH_4_ is given in Table 1.

**Table 1:** Comparison of the productions starting from either [^11^C]CO_2_ or [^11^C]CH_4_

Parameter	Method 1 ([^11^C]CO_2_)	Method 2 ([^11^C]CH_4_)
Number of syntheses	94	35
Yield in GBq (EOS)	2.6 ± 1.3 GBq	0.76 ± 0.27 GBq
Yield in % (decay-corrected to [^11^C]CH_3_I)	13.4 ± 6.2%	16.1 ± 4.9%
Molar activity (EOS)	88 ± 57 GBq/μmol	157 ± 68 GBq/μmol
Irradiation time	30–40 min	25 min
Overall synthesis time (EOB)	approx. 35 min	approx. 34 min

*EOS* end of synthesis, *EOB* end of bombardment

## Discussion

For clinical use of [^11^C]erlotinib in patients a stable and reproducible radiosynthesis is essential. So far, four different synthesis procedures of [^11^C]erlotinib have been published employing different solvents (DMF or CH_3_CN), reaction temperatures (80 °C or 120 °C) and bases (NaH and TBAH) for the deprotonation of the alcohol functionality of the precursor (Table 2). The highest, but also most fluctuating molar activities have been reported by Bahce et al. (2013) (Table 2). However, the [^11^C]CH_3_I production method is not fully specified in the work by Bahce et al. (2013), which is an important factor influencing molar activity. The two studies which used NaH as base for the synthesis of [^11^C]erlotinib (Memon et al. 2009; Petrulli et al. 2013) did not state radiochemical yields. We employed the reaction conditions of Bahce et al. (2013) for setting-up the radiosynthesis of [^11^C]erlotinib in our laboratories. The usage of CH_3_CN as solvent and TBAH as base led to final product amounts of 2.6 ± 1.3 GBq (method 1) or 0.76 ± 0.27 GBq (method 2), which was considered sufficient for clinical and preclinical use of the radiotracer, so that no further optimization of synthesis parameters was performed. Method 1 afforded higher final product amounts than method 2, due to the possibility to produce higher starting activities with the [^11^C]CO_2_ target. The employed starting activities for the [^11^C]CH_4_ target were 27–43 GBq [^11^C]CH_4_ for an irradiation time of 12–22 min. For a clinical use the irradiation time for [^11^C]CH_4_ production could be potentially prolonged, providing a maximum possible starting activity of approximately 70 GBq (EOB). In order to compensate the time loss due to the reduction step of [^11^C]CO_2_ in method 1, the flow rate in the semi-preparative HPLC purification was increased from 2.5 mL/min (method 2) to 8 mL/min (method 1). Precursor and product [^11^C]erlotinib were still sufficiently separated with the increased flow rate and eluted with retention times of 2.5–3.5 min and 4.5–5.5 min, respectively (Fig. 3a). The shortening of the HPLC purification time in method 1 led to comparable total synthesis times for both methods. The [^11^C]CH_4_ method afforded higher molar activities of [^11^C]erlotinib. This can mainly be attributed to the fact that atmospheric CO_2_ is ubiquitous and can contaminate the radiosynthesis, which lowers molar activity in method 1. Andersson et al. (2009) reported 7–14-fold improvements in molar activities of four different PET tracers by using [^11^C]CH_4_ as compared with [^11^C]CO_2_. Such high increases in molar activity were not obtained in our work (1.8-fold increase in molar activity with [^11^C]CH_4_), which may be related to other unknown differences in synthesis set-ups or impurities in the employed chemicals. For other [^11^C]tracers synthesized in our laboratory (e.g. [^11^C]DASB), the presently employed set-up (method 2) afforded molar activities at EOS up to 1 TBq/μmol. Additional optimization included the setup of an ultra-HPLC system, which reduced the time of the RP-HPLC run of the quality control from 10 to 1 min. (Nics et al. 2018) This reduction in time for the quality control would afford a 27% increase in molar activity at the time of PET tracer administration into a patient, as compared to use of the conventional HPLC system. To ensure that no radioactive impurity is missed, we compared the optimized HPLC method to the conventional assay: all peaks (including impurities), which were detected in the conventional system could also be detected in the same ratios in the improved set-up.

**Table 2:** Comparison of synthesis procedures described in literature

Literature	[^11^C]CH_3_I Production method	Reaction time	Solvent	Temperature	Base	Molar activity	Radiochemical purity	Yield
Memon et al. 2009	not specified	5 min	DMF	120 °C	NaH	20–100 GBq/μmol	<95%	n.a.
Bahce et al. 2013	not specified	5 min	CH_3_CN	80 °C	TBAH	184–587 GBq/μmol	>98%	2.18–3.48 GBq
Petrulli et al. 2013	[^11^C]CO_2_ via GE FX MeI module	5 min	DMF	120°	NaH	159 ± 48 GBq/μmol	>99%	n.a.
Slobbe et al. 2015	[^11^C]CO_2_ via LiAlH_4_	5 min	DMF/CH_3_CN	120 °C	TBAH	287 ± 63 GBq/μmol	>99%	13.1 ± 3.7%*

*n.a.* not available

* Corrected for decay

Abourbeh et al. (2015) reported an inverse correlation between [^11^C]erlotinib uptake in HCC827 tumor xenografts and injected carrier mass of unlabelled erlotinib, suggesting that saturation of EGFR-specific binding of [^11^C]erlotinib occurred. Similarly, Bahce et al. (2013) reported a reduction in tumoral volume of distribution (V_T_) of [^11^C]erlotinib, when NSCLC patients underwent [^11^C]erlotinib PET scans during erlotinib therapy as compared to PET scans when they were off therapy (Bahce et al. 2016). These data suggest that an increase in molar activity of [^11^C]erlotinib by using the [^11^C]CH_4_ method may be beneficial for improved target-to-non target ratios in EGFR imaging, although a potential disadvantage of method 2 is the lower final product amount. However, a systematic comparison of the influence of molar activity on diagnostic performance of [^11^C]erlotinib has not been performed yet.

## Conclusion

We compared two different methods for the synthesis of [^11^C]erlotinib, starting either from [^11^C]CO_2_ or from [^11^C]CH_4_. Both methods reliably yielded sufficiently high product amounts for preclinical and clinical use. The [^11^C]CH_4_ method yielded 1.8-fold higher molar activities, which may be beneficial for improved target-to-non target ratios in EGFR imaging of tumors. In order to keep the time consumption to a minimum, a highly efficient RP-HPLC method was established lasting for not more than 1 min, which can be expected to lead to an increase in molar activity at the time of radiotracer injection into a patient.

## References

[CR1] Abourbeh G, Itamar B, Salnikov O, Beltsov S, Mishani E (2015). Identifying erlotinib-sensitive non-small cell lung carcinoma tumors in mice using [^11^C]erlotinib PET. EJNMMI Res.

[CR2] Andersson J, Truong P, Halldin C (2009). In-target produced [^11^C]methane: increased specific radioactivity. Appl Radiat Isot.

[CR3] Bahce I, Smit EF, Lubbernik M, van der Veldt AAM, Yaqub M, Windhorst AD, Schuit RC, Thunnissen E, Heideman DAM, Postmus PE, Lammertsma AA, Hendrikse NH (2013). Development of [^11^C]erlotinib positron emission tomography for in vivo evaluation of EGF receptor mutational status. Clin Cancer Res.

[CR4] Bahce I, Yaqub M, Errami H, Schuit RC, Schober P, Thunnissen E, Windhorst AD, Lammertsma AA, Smit EF, Hendrikse NH (2016). Effects of erlotinib therapy on [^11^C]erlotinib uptake in EGFR mutated, advanced NSCLC. EJNMMI Res.

[CR5] Bauer M, Matsuda A, Wulkersdorfer B, Philippe C, Traxl A, Özvegy-Laczka C, Stanek J, Nics L, Klebermass E-M, Poschner S, Jäger W, Patik I, Bakos É, Szakács G, Wadsak W, Hacker M, Zeitlinger M, Langer O. Influence of OATPs on Hepativ disposition of Erlotinib measured with positron emission tomography. Clin Pharmacol Ther. 2017; 10.1002/cpt.888. 10.1002/cpt.888 PMC6083370 28940241

[CR6] Ciardiello F, Tortora G (2008). EGFR antagonists in cancer treatment. N Engl J Med.

[CR7] Cohen MH, Johnson JR, Chen Y-F, Sridhara R, Pazdur R (2005). FDA drug approval summary: erlotinib (Tarceva®) tablets. Oncologist.

[CR8] Larsen P, Ulin J, Dahlstrøm K, Jensen M (1997). Synthesis of [^11^C]iodomethane by iodination of [^11^C]methane. Appl Radiat Isot.

[CR9] Memon AA, Jakobsen S, Dagnaes-Hansen F, Sorensen BS, Keiding S, Nexo E (2009). Positron emission tomography (PET) imaging with [^11^C]-labeled erlotinib: a micro-PET study on mice with lung tumor xenografts. Cancer Res.

[CR10] Nics L, Steiner B, Klebermass E-M, Philippe C, Mitterhauser M, Hacker M, Wadsak W (2018). Speed matters to raise molar radioactivity: fast HPLC shortens the quality control of C-11 PET-tracers. Nucl Med Biol.

[CR11] Petrulli JR, Sullivan JM, Zheng M-Q, Bennett DC, Charest J, Huang Y, Morris ED, Contessa JN (2013). Quantitative analysis of [^11^C]-erlotinib PET demonstrates specific binding for activating mutations of the EGFR kinase domain. Neoplasia.

[CR12] EDQM - European Directorate for the Quality of Medicines. Radiopharmaceutical Preparations. (Radiopharmaceutica, 8.0/0125). European Pharmacopoeia. 8th ed. Vienna: Official Austrian Version, Verlag Oesterreich GmbH; 2008. p. 1167–73.

[CR13] Singh M, Jadhav HR (2017). Targeting non-small cell lung cancer with small-molecule EGFR tyrosine kinase inhibitors. Drug Discov Today.

[CR14] Slobbe P, Windhorst AD, Stigter-van Walsum M, Smit EF, Niessen HG, Solca F, Stehle G, van Dongen GAMS, Poot AJ (2015). A comparative PET imaging study with the reversible and irreversible EGFR tyrosine kinase inhibitors [^11^C]erlotinib and [^18^F]afatinib in lung cancer-bearing mice. EJNMMI Res.

[CR15] Traxl A, Komposch K, Glitzner E, Wanek T, Mairinger S, Langer O, Sibilia M (2017). Hepatocyte-specific deletion of EGFR in mice reduces hepatic Abcg2 transport activity measured by [^11^C]erlotinib and positron emission tomography. Drug Metab Dispos.

